# Integration of a tele-dermatology model for Epidermolysis Bullosa improves patient outreach: a before and after observational study

**DOI:** 10.3389/fdgth.2026.1848667

**Published:** 2026-06-26

**Authors:** Aditya Viswanath, Divya Gupta, Manoj Srinivasa, Tim Jose, Silji Jibins, Charles Mendith, Utsav Munnoli, Asha Jubba, Guruduta Baraka, Ravi Hiremagalore

**Affiliations:** 1 Leicester Medical School, University of Leicester, Leicester, United Kingdom; 2 Department of Dermatology, Dr. B R Ambedkar Medical College and Hospital, Bengaluru, India; 3 Centre for human Genetics, Bengaluru, India; 4 Department of Dermatology Sri Madhusudan Sai Institute of Medical Sciences and Research, Chikkaballapur, Karnataka, India; 5 Centre of Human Genetics, Bengaluru, Karnataka, India; 6 Dermatopathologist, Delhi Dermath Laboratory, New Delhi, India; 7 Department of Human Genetics National, NIMHANS, Bangalore, India; 8 Department of Dermatology, University of Alabama, Birmingham, AL, United States

**Keywords:** accessible care, cost-analysis, digital health, epidermolysis bullosa, global health, India, rare disease, tele-dermatology

## Abstract

**Background:**

A hub-and-spokes model strengthens care for Epidermolysis Bullosa by combining specialized expertise with community clinics. Genetic and genomic diagnostics power personalized treatment, whilst telemedicine improves access and affordability of patient care. This integrated system delivers equitable and patient-centered care, reducing burden and improving outcomes for individuals with rare genetic diseases.

**Methods:**

This was a before-and-after observational study conducted at the Centre for Human Genetics in Bangalore, between January 2020 and August 2025. Patients with confirmed or suspected Epidermolysis Bullosa (EB) were referred, either by spoke-level practitioners or family members, to a teleconsultation with specialized dermatologists. Clinical data, including family history, were securely documented, and a multidisciplinary team was engaged when further intervention was required. Continuity of care was facilitated through a HIPAA-compliant mobile application, enabling families to maintain communication and follow-up. The impact of the program was evaluated by analyzing its geographical reach, patient evaluation, and comparative medical costs before and after implementation.

**Results:**

Developing a standardized diagnostic framework and care pathways for the heterogeneous genetic condition Epidermolysis Bullosa (EB) across a large geographic region addressed a critical gap in the healthcare system. Within our program, 271 patients were enrolled, comprising EB simplex (*n* = 67), junctional EB (*n* = 69), dystrophic EB (*n* = 104), and Kindler syndrome (*n* = 7). Geographical data, available for 41 patients, demonstrated that most (*n* = 31, 75.6%) resided within a 600 km radius of the central hub, while the most distant patient was located 3125 km away, underscoring both the initiative's reach and accessibility. Importantly, implementation of the program resulted in a statistically significant reduction in direct medical costs, with a median savings of Rs 2625 (IQR: −1212–9025), as confirmed by a two-tailed Wilcoxon signed rank test (W = 143, *n* = 32, *P* = 0.0225). These findings highlight the feasibility and impact of centralized diagnostic and care models in improving access and reducing the economic burden for patients with EB.

**Conclusion:**

Patient care for Epidermolysis Bullosa has focused beyond economic benefits; the model demonstrates how technology can enhance equity in healthcare delivery. Telemedicine and genomic technologies bridge critical gaps in specialized care, particularly for complex conditions such as EB across wide geographic regions. A centralized diagnostic and care framework across wide geographic regions allows for statistically significant reductions in direct medical costs

## 1 Introduction

Epidermolysis Bullosa is a group of inherited skin disorders of mucosal fragility. The disease stems from mutations that alter the structural integrity of the epidermal layer and components attaching the epidermis to the dermis (1). EB is grouped into four major subtypes: Simplex (EBS), Junctional (JEB), Dystrophic (DEB), and Kindler Syndrome (KS). EBS is the mildest form, with blisters and superficial erosions, causing little morbidity, whilst JEB is very severe and mostly fatal. Recessive Dystrophic EB can be fatal or have severe morbidity resulting from extracutaneous involvement, such as fused hands and feet (mitten-hand deformity), gastrointestinal involvement with feeding difficulty that leads to nutritional deficiency, as well as limbal stem cell deficiency in the eyes, endocrine abnormalities, and early osteopenia (2).

Epidemiological data for EB show significant variation between nations. High incidence and prevalence rates are reported amongst developed nations such as the UK, Netherlands, and Germany (3–5). Low and Middle Income countries (LMICs) lack such national registries; in India, despite advances in diagnosis and management, significant gaps persist in understanding both the true prevalence of EB and the preparedness of health systems to address the complex requirements of this rare genetic condition (6). Focused initiatives that integrate epidemiological research, specialized clinical expertise, and resource-appropriate care are essential to bridge these gaps (7). Strengthening EB-centric programs will not only improve patient outcomes but also enhance system readiness, ensuring that individuals affected by this heterogeneous disorder receive equitable, comprehensive, and sustainable care.

Management is multifaceted, involving wound care, surgical, and psychosocial intervention by the multidisciplinary team (MDT). High disease burden has been reported both through the lens of the patient and the caregiver (8, 9). Studies from the USA and UK further demonstrate a significant financial burden for patient families, owing to dressing costs, specialist visits, and supportive therapies (10, 11). LMICs lack formal cost analysis, although it is logical to assume that financial barriers limit access to specialized care, worsening clinical outcomes. Accessibility and affordability remain major barriers to care for patients with EB. Many live far from specialized centers, face limited availability of trained experts, and struggle with high out-of-pocket costs and inadequate financial resources. Health facilities often lack infrastructure, leading to delayed diagnoses and fragmented continuity of care, whilst limited awareness among patients and providers further restricts timely intervention. Addressing these challenges is essential to ensure equitable and sustainable care delivery. Tele dermatology has emerged as a transformative model of healthcare, and its diagnostic reliability and treatment efficacy has been shown to be comparable to in-person consultation (12). In the context of Epidermolysis Bullosa, tele-dermatology models can optimize disease education and facilitate MDT discussion and management.

Therefore, we proposed to evaluate a tele-dermatology driven initiative, which ran from 2020 to 25, designed to improve access to specialized epidermolysis bullosa care in rural areas of India, with a central hub located in Bangalore, Karnataka. Through assessing geographical reach, cost-effectiveness, and patient response, we sought to assess the viability of a digital intervention for deprived groups with a rare skin disease. We hypothesize this technology-enabled hub-and-spokes model would make EB care more accessible and reduce the financial burden of the disease on patients and their families.

## 2 Methods

### 2.1 Study participants and ethics

**Figure 1:**
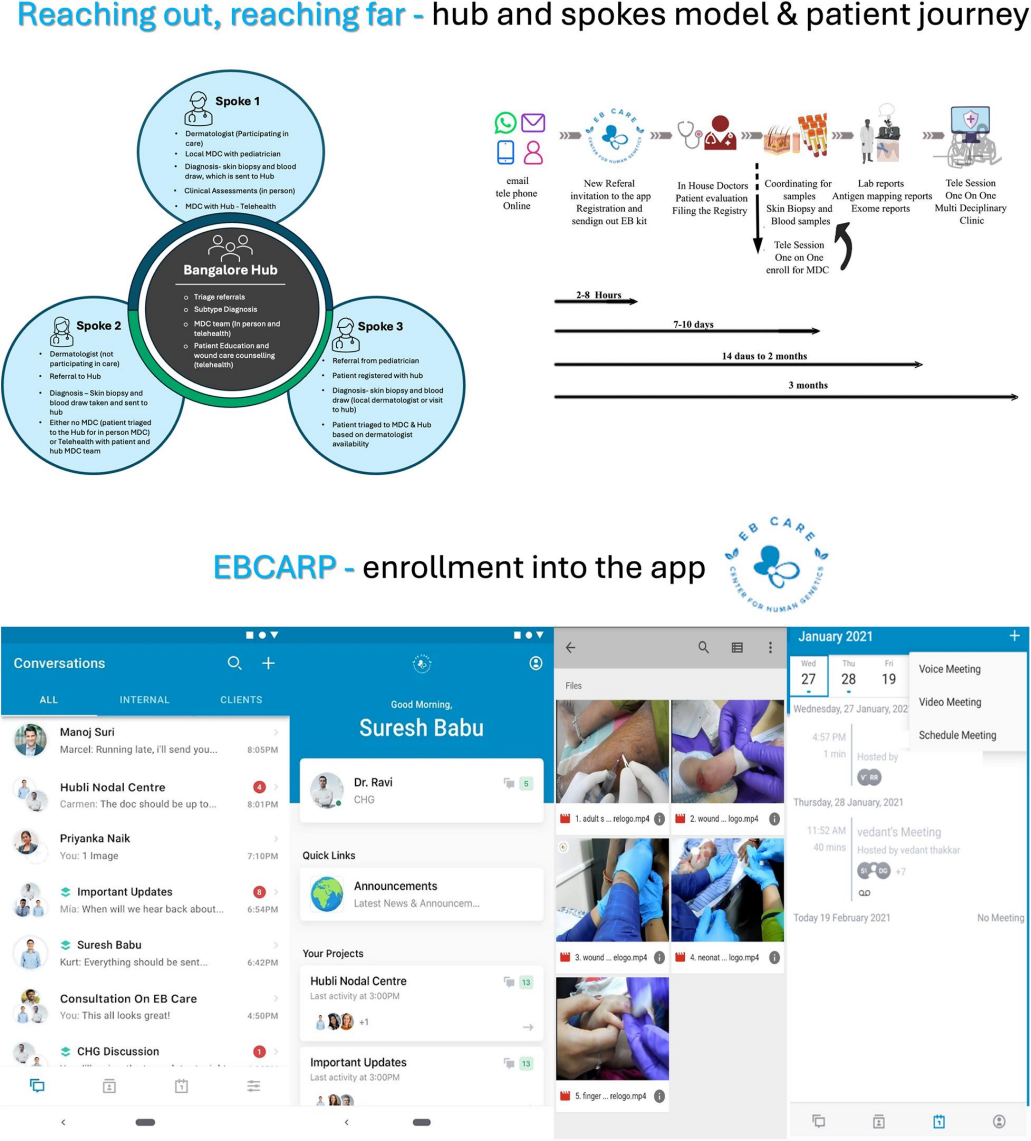
**(A)** schematic representation of the tele dermatology workflow. The left Hub and spokes model describes the procedural components for the Hub at Bangalore, and each spoke, highlighting which aspects were telehealth vs. in-person based (MDC – multidisciplinary clinic). The right diagram illustrates the sequential process from patient referral up until remote teleconsultation. Arrows indicate the progression through this process. **(B)** Overview of the digital platforms used in the tele-dermatology program, including representative screenshots of the application interface used for patient data entry, image sharing, and clinician interaction. Referral link (https://chgregistryapp.appspot.com/register/chg).

This is an observational study evaluating a hub and spokes tele dermatology model of care for Epidermolysis Bullosa. The study was conducted between 2020 through to 2025 at the Centre for Human Genetics, Bangalore, India, and recorded 271 patients during this period. Centre for Human Genetics is a nationally recognised referral and research center for inhertied genetic disorders. The centre has established facilities for comprehensive clinical phenotyping, molecular diagnostics including next-gen sequencing, functional genomics suppoirted by Clinical geneticists, dermatologists, and scientists. Comprehensive Care in Epidermolysis Bullosa is an initiative of the Division of Dermatology started in 2012. A registry was established in 2020 with generous funding from Epidermolysis Bullosa Research Partnership (EBRP). The program focuses on diagnosis and comprehensive care for patients with EB. Centre of Human Genetics (CHG) served as the central hub, providing diagnostic facilities and coordinating telehealth care. Dermatologists and pediatricians in several regions across the state of Karnataka were designated as ’spokes’ and were invited to refer patients with suspected or diagnosed EB to our service using a secure referral link (Figures 1A, B). This generated an email to the EB clinic coordinator. The referring physician was then contacted to decide their role in care. Interested physicians established a basic MDT locally with the guidance of the team at Bangalore. In the case where the referring physician did not want to be involved, the team at Bangalore provided care. The study was approved by the local ethics committee at CHG (Ref No CHG/077/2020-21/001-07/2020).

### 2.2 Model of consultation, diagnosis, and management

Delivery of care was through both synchronous (telehealth consults) and asynchronous (store-and-forward images) methods. Initial consults were typically conducted synchronously, except in clearly straightforward cases. Follow-up care was predominantly asynchronous, except in more complex cases. This was done using a SOC 2 and HIPAA compliant app, designed as a tool for communication between patients and the providers at CHG, called Epidermolysis Bullosa-Care and Registry Program (EB-CARP) (Moxo Solutions, version 9.5, Cupertino, USA) (Figure 1B). The app was free to download on a basic Android smart phone, and after receiving the referral, patients were invited to join. Each patient had at least one initial in-person or telehealth consultation with an EB-trained dermatologist. During this consultation, a detailed history including medical, family, and social were obtained, and photographs of the affected regions of the skin were submitted by the referring dermatologist or pediatrician. Subtype of EB was confirmed through exome sequencing in all patients, and immunofluorescence on skin biopsies in patients who were infants, or where clinical manifestations overlapped between different subtypes. Patients were then enrolled in the registry, and all documents, including those shared on the app, were uploaded alongside the medical history onto ‘Docpulse’, a cloud-based, HIPAA compliant healthcare software platform. Patients were then triaged to the multidisciplinary clinic by the nurse, based on clinical manifestations. All patient families were educated about the condition, prognosis, and genetic counseling including prenatal testing. Focus on wound care and interventions were planned if needed, based on the recommendations of the MDT. Wound dressings were initially provided, and therein after subsidized by the program in accordance with the patient's needs. In-person referrals to the ‘hub’ were done only if care could not be established locally. Follow-up of patients was based on their needs, and was accomplished by the app.

### 2.3 Evaluation of a Hub and spokes model of care using teleconsultations

The impact of the “Hub and spokes model” was evaluated through geographical distance of the patient from the hub, changes in patient reported care costs, and a questionnaire to assess the quality of the teleconsultation and the program itself. The data was collected either at the end of the consultation or via telephone conversation at follow-up. Distances covered were recorded as the actual distance in kilometers between the patient's resident city to Centre of Human Genetics, Bangalore. The medical costs were recorded, when possible, on the initial consult and at the most recent follow up. Direct medical costs included the cost of prescriptions, consultations, investigations, and in-hospital care per month, and indirect medical costs included loss of wages incurred by families from having to take time off to attend medical appointments and providing care for the patient, along with the cost of travel, food, and accommodation for visits to specialized centers of care. The questionnaire included the quality of audio, video, and the utility of the telehealth-based approach as opposed to an in-person type system, with responses rate on a 1–5 scale, with 5 indicating the highest quality level.

### 2.4 Statistical analysis

Analysis of sociodemographic data was conducted using Microsoft Excel (V16.102). For cost changes, a Wilcoxon signed rank test was used to assess the significance of the paired cost data change. The test was performed using statistical software Prism GraphPad (V10.6.1). The median of the paired differences was then reported as the ‘median change’. Publicly available mapping software was used to create a visual reflection of patient spread.

### 2.5 Role of the funding source

Epidermolysis Bullosa Research Partnership is a non-profit organization aimed to fund EB related activities and research. The authors recognize their generous funding which helped to build a robust platform to capture and store data, which is critical for the registry. It also helped to fund the app for communications and the generation of genotype data. EBRP was not involved in idea generation, data collection, drafting, and writing of the manuscript.

## 3 Results

### 3.1 Socio-demographic profile, enrollment, and clinical evaluation of EB patients

A total of 271 individuals with Epidermolysis Bullosa were recognized, referred, and consulted through this app-based tele-dermatological initiative. The median age was 0·88 years [interquartile range (IQR) 0·1–8·3]. Of the total cohort, 149 (55%) were male, and 122 (45%) were female.

**Figure 2:**
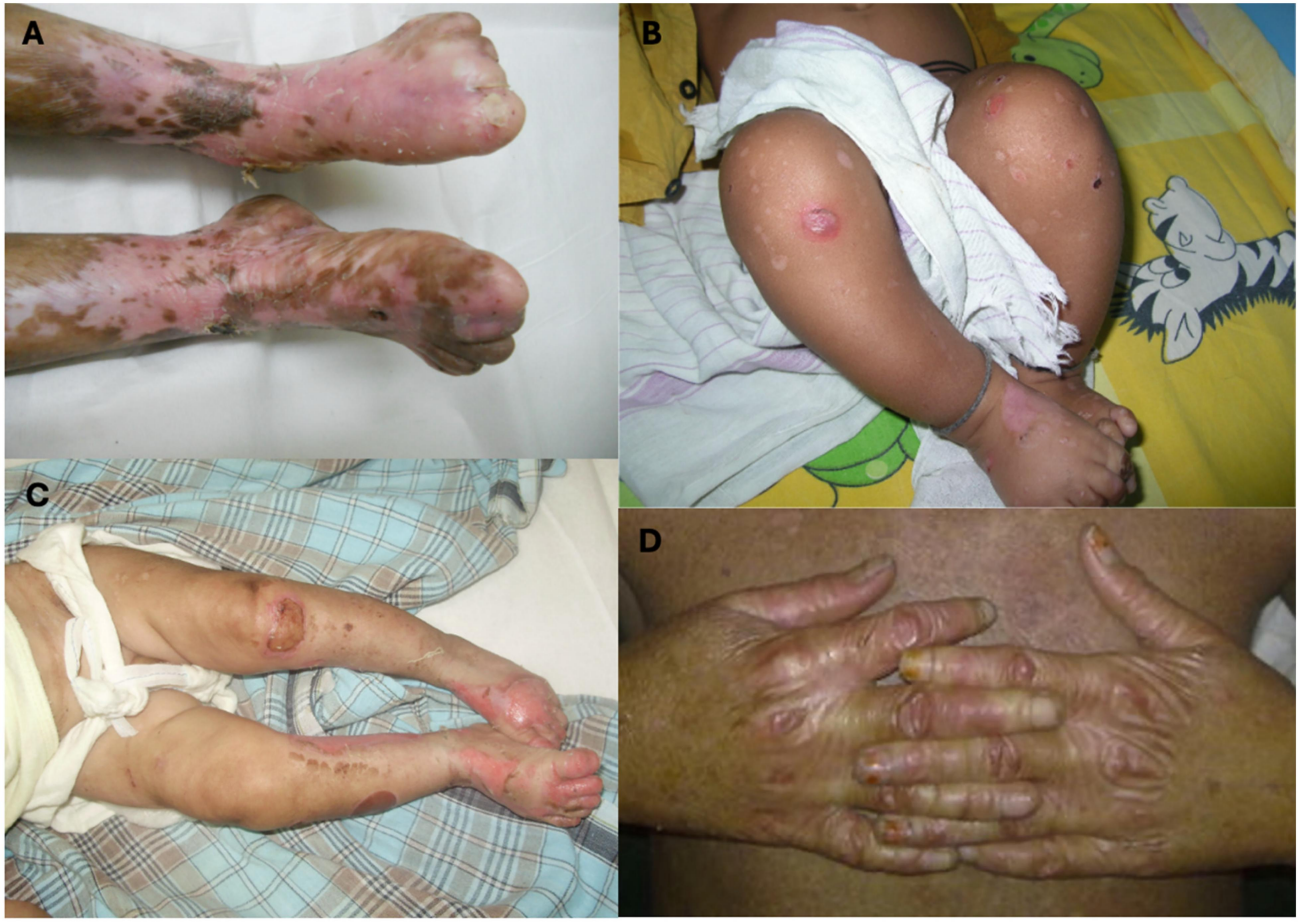
Representative manifestations of epidermolysis Bullosa subtypes. **(A)** Dystrophic EB demonstrating extensive scarring, erosions, and pseudo-syndactyly of the feet. **(B)** EB simplex showing localised blistering. **(C)** Junctional EB with erosions affecting the lower limbs. **(D)** Kindler syndrome demonstrates cutaneous atrophy and wrinkled skin (poikiloderma) over the trunk and hands.

EB subtype was documented in 247 (91·1%) of patients. This was based on clinical phenotyping, skin biopsy, and genetic sequencing. Among these, 67 (24·7%) were classified as Simplex, 69 (25·5%) as Junctional, and 104 (38·4%) as Dystrophic. Kindler syndrome was recorded in 7 (2·6%). Given the high prevalence of consanguinity, we found the recessive forms of EB accounting for almost half the cases. Baseline characteristics at first visit were recorded; data completeness varied by domain, and the number of patients with available data is indicated as (n) for each. Oral mucosal lesions were present in 107 (51·0%, *n* = 210), eye involvement in 31 (14·5% *n* = 214), nail involvement in 140 (63·3% *n* = 221), Genitourinary mucosal involvement in 24 (11·7% *n* = 205), and hair loss in 28 (15·5% *n* = 181). Sociodemographic data and disease classification are summarized in Table 1. Clinical Images are shown in Figure 2.

**Table 1:** Sociodemographic and clinical characteristics of the epidermolysis Bullosa cohort.

Sociodemographic/Clinical Characteristics	Value *n* (%)
Sociodemographic	
Total Patient no.	271
Age at first consult^a^	0.9 (0·1–8·3)
Sex (Male)	149 (55)
EB subtype	247 (91·1)
Simplex	67 (24·7)
Junctional	69 (25·5)
Dystrophic	104 (38·4)
Kindler	7 (2·6)
Missing	24 (8·9)
Oral Mucosal Lesions, *n* (210)	107 (51)
Ocular Involvement, *n* (214)	31 (14·5)
Nail Changes, *n* (221)	140 (63·3)
Hair Loss, *n* (205)	24 (11·7)
Genito-mucosal Lesions, *n* (181)	28 (15·5)

Data presented as *n* (%) unless otherwise indicated.

a Value reported as Median (Interquartile range).

**Figure 3:**
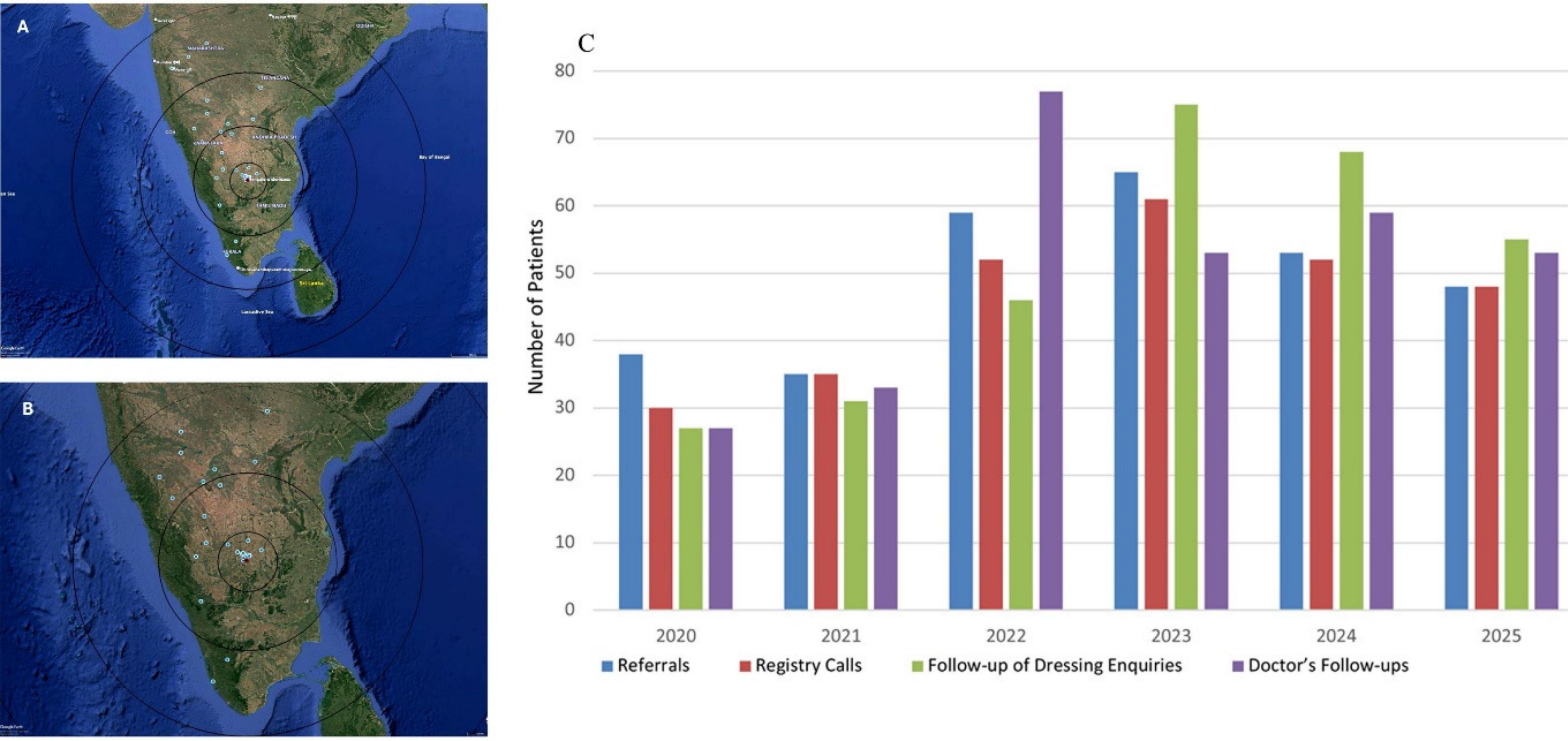
Geographic distribution of patients accessing the program. **(A)** Regional Distribution of patients with distance rings centered on the programmed hub at 100, 300, 600, 1000 (outermost) km. **(B)** Mid-range view showing patients at 600 km (outermost) from the hub blue dots represent Patient Locations, the red marker represents the central Hub, CHG, Bangalore. **(C)** Tele dermatology referral and follow up activity, 2020 – (August) 2025. Annual Counts are shown for referral calls, register entries, dressing enquiries, and doctor follow ups. Data recorded for 2025 included activity from January to August.

A total of 443 consultations were undertaken. Referral information, as well as follow up of dressing enquiries and doctor-led follow-ups are represented in Figure 3C. This figure excludes nurse lead consults, which were conducted separately for the purpose of wound care and patient education.

### 3.2 Geographic reach of Hub-and-spoke care for EB patients

Geographical data was recorded for 41 patients across 8 Indian states, with patients primarily concentrated in Karnataka, but also extending to remote regions of West Bengal and Assam.

For 34 (82·9%) of 41 patients, geographical identifiers (state and city) were recorded, with the remaining 7 having recorded state only, or vague region only. The farthest patient was located approximately 3125 km from the central hub. Of the cohort, 13 (31·7%) of patients were located within a 100 km radius of the central hub, 7 (17·1%) in 100–300 km radius, 11 (26·8%) within 300–600 km, 4 (9·8%) within 600–1000 km, 4 (9·8%) within 1000–1500 km, and 2 (4·9%) outside of 1500 km radii (Figures 3A, B).

### 3.3 Economic burden and cost implications of epidermolysis Bullosa care

To assess the financial impact of the intervention, medical costs per month were documented for up to 33 patients before and after enrolling in this tele-dermatological program. Cost analyses were split into two groups based on data availability.

The first group (*n* = 33) included patients with complete data on direct medical costs. One patient in this group was excluded from the final analysis due to an implausible discrepancy between their pre and post intervention costs, likely reflecting an error in documentation. Among the 32 remaining patients, the median monthly cost prior to the program entry was Rs 6,500 (IQR 2,500–12,775), falling to Rs 3125 (IQR 1875–6104) following the intervention. The resulting median monthly saving was Rs 2625 [IQR (-) 1212–9025]. A Wilcoxon signed rank test indicated the reduction was statistically significant [W = 143, *N* = 32, *p* = .0225 (two-tailed)]. Cost reductions were observed in 20 of the 32 participants (62.5%). When contextualized against publicly available estimates of average income in Karnataka (Rs 36,000), this corresponds to approximately 7% of monthly earnings (13).

**Figure 4:**
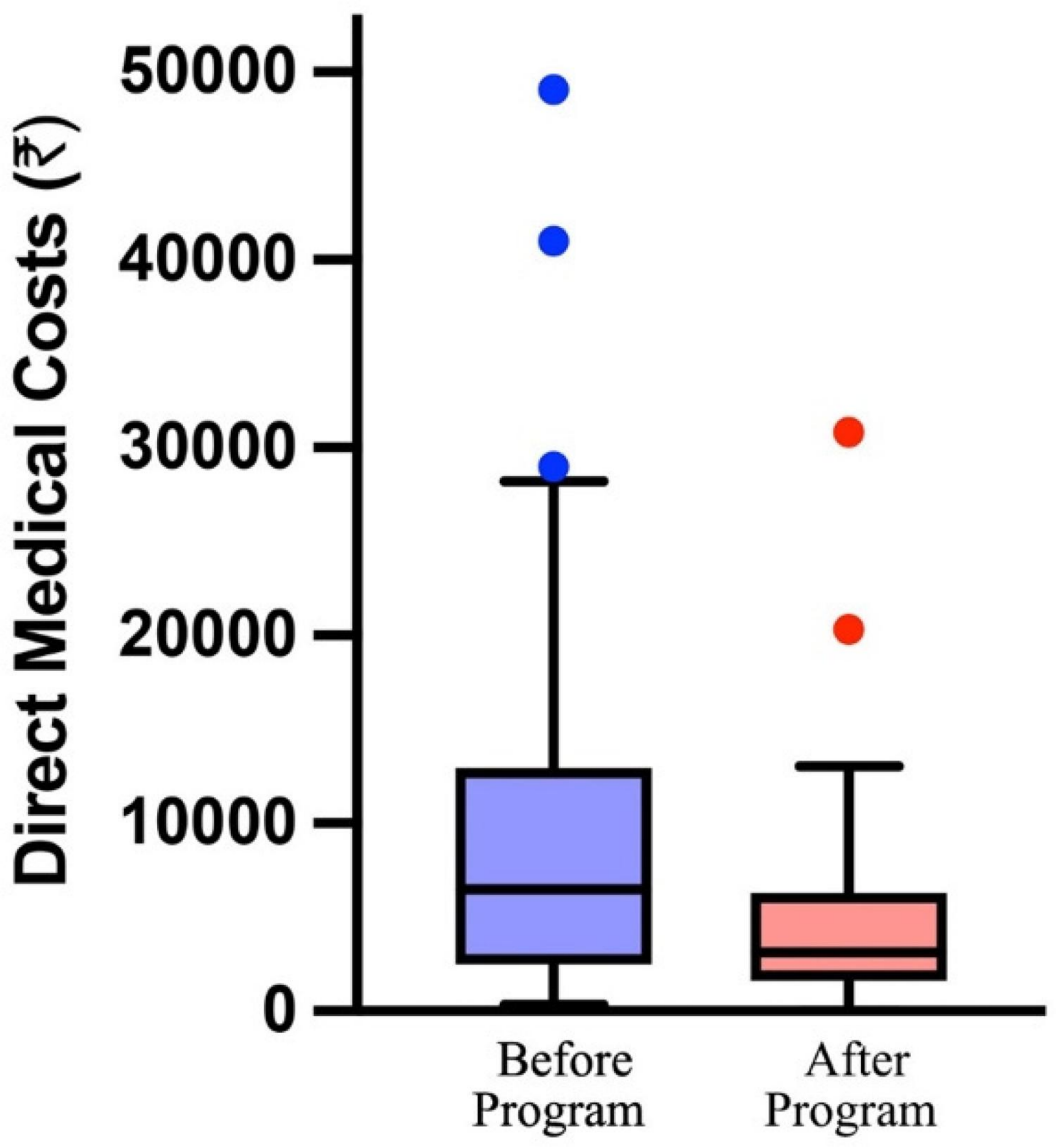
Direct medical costs before and after the tele dermatology program. Tukey box and whisper plot shows the distribution of direct medical costs before and after the programme’s implementation. Boxes represent the IQR, horizontal lines, indicate the median, whiskers show Tukey Limits, and points denote outliers. Costs are presented in Indian Rupees.

A secondary analysis was performed in a subgroup of 28 patients with complete data on both Direct and Indirect medical costs, the latter being self-reported productivity losses. In this group, the median monthly cost prior to enrolling into the program was Rs 11,250 (IQR 4500–22,000), which reduced to Rs 4830 (IQR 1875–7536) after the intervention. The median monthly saving was Rs 4,042 [IQR (-)572–16,6678]. This reduction was also of statistically significant [W = 62, *n* = 28, *p* < .001 (two tailed)]. Twenty patients (71.4%) reported net cost savings. Findings are illustrated using a Box and Whisker plot (Tukey) in Figure 4.

### 3.4 Evaluation

Patient and family reported evaluation was available for up to 30 patients across evaluation domains. Of those, the rating of video quality scored a median of four [IQR 3–5, 5/5 (38%), 4/5 (35%)], audio quality with a median of five [IQR 4–5, 5/5 (63%)], and overall perception of the session scored a median of 5 (IQR 4–5).

## 4 Discussion

This study demonstrates the impact of a tele-dermatology program for Epidermolysis Bullosa (EB) in rural areas of Karnataka and other states in India, implemented through a hub-and-spokes model anchored at the Centre for Human Genetics, Bangalore. A total of 271 patients across diverse EB subtypes were successfully enrolled, underscoring the program's ability to reach a heterogeneous population. The findings highlight the capability of telemedicine to extend specialized care to geographically dispersed communities, thereby addressing one of the most critical barriers in rare disease management - accessibility. With most patients residing within a manageable distance of the hub, and some located thousands of kilometers away, the model demonstrated its potential to bridge gaps in healthcare delivery. Importantly, beyond improving access, the program resulted in statistically significant financial savings for families, reinforcing its economic viability and sustainability in resource-limited settings. To our knowledge, this is the first observational study globally to evaluate the adaption of a dedicated tele dermatology program for patients with EB in an LMIC.

Whilst tele-dermatology has been widely implemented globally, with its use accelerated by the COVID-19 pandemic, most studies are represented in high-income or urban settings (12, 13). The evidence consistently supports its diagnostic reliability, acceptance by patients, and support of its use in LMICs (12, 14). However, its application to rare dermatological disease such as epidermolysis bullosa remains unexplored, with existing literature limited to descriptions and recommendations of its use during the COVID pandemic (15). EB care in India remains inaccessible, unaffordable, and unavailable due to systemic gaps in healthcare delivery (6, 7). Our study demonstrated improved geographical access to specialist care for EB in rural India, consistent with findings from the literature (16).

A similar initiative conducted in Indonesia by Adella et al. implemented a WhatsApp-based service for a broad range of dermatological diseases in a limited resource setting (17). Whilst both studies committed to improving geographical access for patients, our study focused on a rare genetic disorder, using real-time consultation as opposed to solely store-and-forward methods. Furthermore, whereas the Indonesian study prioritized education to general health workers to build diagnostic competence, our intervention provided EB-specific specialist support through MDT planning. These differences highlight the importance of tailoring telehealth models to disease specific contexts and local care systems, ensuring interventions meet the needs of the patient. Additional insights from the international Rare Disease Research Consortium (IRDiRC) task force emphasize the critical role of telehealth in overcoming specialist scarcity for a multitude of rare diseases (18). Our study uniquely adds to the insights of this review by addressing the domain of specialist care for EB. Moreover, through analysis of geographical and economic factors of telehealth, we address an evidence gap identified in the IRDiRC review regarding the quantitative impact on health economics of such telehealth initiatives.

Mobile application based tele-dermatology has been increasingly explored as a method of improving access to care. Prior work has demonstrated the feasibility of app-based assessment, though typically relying on asynchronous image submission within the context of automated or AI driven analysis (19). Notably, such approaches are often assessed within higher-income regions and may not address the logistical barriers presented by LMICs (19). Contrastingly, the present study describes the implementation of a hybrid (synchronous-asynchronous) model within a real-world, resource-constrained setting. Rather than relying on AI automated analysis, the app emphasizes clinically led decision making, enhancing reliability and acceptability. This is especially important in rare disease context, such as EB. Therefore, whilst emerging digital literature place primary focus on automated workflows, scalable improvements, especially in the context of a rare disease within LMICs, still must rely on pragmatic models such as the one used in the present study.

The financial burden of Epidermolysis Bullosa on patients and families has been well documented in resource-plentiful areas. A UK based study examining recessive dystrophic EB reported high costs for wound care and dressings, compounded by financial strain due to unemployment, related to the severity of the disease (11). Dystrophic EB consistently demonstrates higher rates of financial burden as compared to milder forms (9). A Spanish study found that direct and indirect medical costs accounted for only 17.2% and 11.5% of total costs, respectively, with 71.3% of costs related to direct non-medical, such as informal care-giving, a parameter we did not measure as part of this study (20). This trend of high financial burden is also seen in LMICs, although its documentation is limited (21). Our study demonstrates that the implementation of a tele dermatology program for EB in rural regions of India is associated with statistically significant cost savings for patients and families, aligning with prior research (22, 23). This was for both direct medical expenses and total medical expenditure (direct + indirect medical), though results in the latter were cautiously interpreted as there was deemed an increased risk of recall bias and contextual variability in indirect costs (e.g., changes in employment and care giving). Importantly, the observed cost savings represent approximately 7% of the average monthly income (13). However, per capita estimates may overrepresent disposable income at the patient level, particularly within rural regions. As such, the financial impact of these savings is likely greater in real-world settings, suggesting tele dermatology interventions may provide significant economic relief amongst underserved populations. Similar studies of mHealth (mobile based technologies) carried out in rural locations of India and Bangladesh, for neonatal and maternal health management, found consistent reductions in costs for patients and families (24–26). Whilst these studies focused on obstetric care and used different interfaces, they highlight the broader viability of mobile based interventions in rural resource-limited areas, where specialist availability is lower. Similarly, our study highlights that the use of a hub and spokes model could address accessibility issues and comprehensive care under a centralized team more uniformly.

This study has several strengths. It is the first to evaluate a dedicated tele dermatology programme for patients with Epidermolysis Bullosa in rural areas, addressing a critical gap in the literature for this rare disease. Furthermore, through the assessment of multiple parameters, we provide a more comprehensive understanding of the intervention's effect on patient care. Telemedicine offers intangible benefits for patients with genetic diseases that extend beyond clinical outcomes. It reduces anxiety by ensuring timely access to specialists, empowers families through education and awareness, and fosters social inclusion by minimizing stigma and isolation. By bridging urban-rural divides, telemedicine promotes equity in healthcare and ensures continuity of care across distances. Caregivers benefit from reduced travel burdens and emotional reassurance, while communities gain stronger support networks and improved health literacy. Together, these intangible advantages create a holistic, sustainable model of care that enhances quality of life for patients with rare genetic disorders like Epidermolysis Bullosa.

This study has several limitations. The quality and completeness of pre-intervention costs were noted to be suboptimal, limiting the cohort size used for analysis; however, the sample size was reasonable for a rare disease such as EB. This limitation may have led to an actual underestimation of the potential patient cost savings, particularly given that values which were documented as 0 rupee in pre-intervention costs were included to avoid bias, even when the post-intervention cost for the same patient showed a positive value, which was deemed an unlikely occurrence. Notably, we did not include direct non-healthcare costs, such as informal caregiving expenses, which constitute a substantial proportion of the financial burden. Furthermore, the observational design may subject this study to unknown confounding factors, such as changing jobs, earnings, care situations, living and variable access to devices, which can have impacts on indirect medical costs. We also acknowledge the importance of a subgroup analysis differentiating economic benefits between the different EB variants, however, due to missing data on interventions, such as surgery, we decided to generalize the analysis to the cohort.

By establishing a centralized diagnostic and care framework, the program successfully enrolled patients across diverse EB subtypes, extended access across wide geographic regions, and achieved statistically significant reductions in direct medical costs. These findings underscore the potential of telemedicine to bridge critical gaps in specialized care, particularly for complex conditions such as Epidermolysis Bullosa. Beyond economic benefits, the model demonstrates how technology can enhance equity in healthcare delivery. Future initiatives should focus on strengthening continuity of care, evaluating long-term clinical outcomes, both clinician- and patient-reported outcomes, and exploring scalability to broader populations, thereby ensuring sustainable improvements in patient care and health system efficiency, particularly in rare diseases. Correlating outcomes with costs, both direct and indirect medical and non-medical, will be more meaningful parameters to be considered for large-scale implementation across different geographical populations and other rare diseases in general.

## Data Availability

The raw data supporting the conclusions of this article will be made available by the authors, without undue reservation.
